# Gastrointestinal Phytobezoars in Small Animals: A Retrospective Study of 18 Cases

**DOI:** 10.3390/ani16040556

**Published:** 2026-02-11

**Authors:** Giulia Maggi, Francesca Pirgher, Federica Valeri, Domenico Caivano, Maria Chiara Marchesi

**Affiliations:** Department of Veterinary Medicine, University of Perugia, Via San Costanzo 4, 06126 Perugia, Italy; francesca.pirgher@gmail.com (F.P.); federica.valeri@dottorandi.unipg.it (F.V.); domenico.caivano@unipg.it (D.C.)

**Keywords:** phytobezoar, foreign body, gastrointestinal disease, endoscopy, enterotomy, dogs, cats

## Abstract

A phytobezoar is a compact mass formed by the accumulation of indigestible food fibers (such as cellulose and lignin) within the stomach and/or intestine. To date, only limited reports describe the clinical management and diagnostic approach to gastrointestinal phytobezoars (GIPs) in dogs and cats. The aim of this study was to describe the clinical features and diagnostic findings obtained through imaging and instrumental investigations in canine and feline cases with GIPs. Medical records of 25 animals diagnosed with GIPs were reviewed, and data on signalment, clinical signs, imaging findings, and follow-up were collected. Seven animals were excluded because the foreign body (FB) consisted of plastic mixed with fibrous material. Eighteen animals (17 dogs and 1 cat) were included: 8 (44.4%) with gastric involvement, 8 (44.4%) with intestinal localization and 2 (11.1%) with both gastric and intestinal localization. The GIP was removed endoscopically in 3 (16.7%) cases, surgically in 7 (38.9%) cases, extracted from the rectal ampulla in 2 (11.1%) cases, and expelled spontaneously through defecation in 6 (33.3%) cases. GIPs are frequently underdiagnosed, as they are associated with nonspecific gastrointestinal signs. Radiography and ultrasonography offer limited diagnostic value, generally suggesting the presence of an FB without definitively confirming it to be GIP. This study contributes to the current literature by detailing the clinical, diagnostic, and interventional features of GIPs in small animals, representing the first report of this condition in veterinary medicine.

## Simple Summary

A phytobezoar is a compact mass formed by the accumulation of indigestible plant fibers. Gastrointestinal phytobezoars (GIPs) in small animals (dogs and cats) are currently considered an uncommon condition, and the available veterinary literature provides limited information regarding their clinical presentation, diagnosis, and management. Medical records of 18 animals diagnosed with GIPs were retrospectively reviewed to evaluate clinical and diagnostic findings. GIPs were identified in various segments of the gastrointestinal tract and were managed using different approaches, including endoscopic removal and surgical intervention, although some cases resolved without the need for treatment. To the authors’ knowledge, this is the first study to describe predisposing factors, clinical and diagnostic features, and therapeutic management of GIPs in dogs and cats.

## 1. Introduction

A bezoar is a compact mass that develops from the accumulation of indigestible materials within the stomach and/or small intestine. Their formation is typically associated with alterations in the anatomy (es. pyloric stenosis or gastric dilatation) or physiology (poor gastric emptying or reduced gastric acidity) of the gastrointestinal tract, combined with repeated exposure to certain ingested substances. Bezoars are classified into several types based on their composition, including trichobezoars (composed of hair or fur) phytobezoars (derived from high-fiber plant material), pharmacobezoars (resulting from medication concretions), and lactobezoars (formed from milk products) [1,2].

Phytobezoars consist of indigestible plant fibers, including cellulose, hemicellulose, lignin, and fruit tannins. In human medicine, excessive consumption of persimmons is a major risk factor for phytobezoar formation. Persimmons are rich in cellulose and tannins, which, in an acidic environment, polymerize into a glue-like coagulum capable of adhering to other gastric contents. Tannins play a central role in phytobezoar formation by acting as cementing agents for indigestible plant fibers, although the precise pathogenic mechanism remains unclear. Factors such as impaired mastication, altered gastric motility, and gastric acidity also appear to contribute to the development of phytobezoars in humans [3,4]. In veterinary medicine, numerous studies have described gastrointestinal phytobezoars (GIPs) in equids and ruminants [5,6,7]. In horses, findings from various studies indicate that persimmon-derived GIPs represent the most common plant-based foreign bodies (FBs). Their presence may lead to a range of clinical signs, from anorexia or weight loss in cases of partial gastrointestinal obstruction to severe colic in animals with complete obstruction. Management of GIP in equids most often involves medical treatment (such as intragastric administration of diet cola) along with dietary modification or, when necessary, surgical intervention [5,6].

To the author’s knowledge, existing reports in the small-animal literature pertain exclusively to trichobezoars in cats, with little documented information on GIP in either dogs or cats [2,8,9,10,11,12]. To date, only one case report has described a dog in which an FB composed of vegetable and plastic material caused intestinal obstruction [12]. This study therefore provides the first detailed report of GIPs in veterinary medicine. The aim of this retrospective investigation is to offer a comprehensive description of the clinical, diagnostic, and interventional features of GIPs in dogs and cats, thereby enhancing the current understanding of this condition in small animals.

## 2. Materials and Methods

A search of the Veterinary Teaching Hospital (VTH) digital database of the University of Perugia was conducted using the following keywords: “phytobezoar,” “foreign bodies,” “plant-derived mass”, “ball of grass”, “endoscopy,” and “enterotomy”. Inclusion criteria for the study required the presence of GIPs, whereas exclusion criteria comprised mixed FBs (vegetal mixed non-vegetal) and cases lacking adequate follow-up information. Digital medical records of 25 animals diagnosed with GIP referred to the VTH of Perugia University between 2019 and 2025 were reviewed.

Data retrieved from electronic medical records included month of presentation, remote and recent medical history, signalment (species, breed, age, sex, weight), clinical signs, findings from physical examination and diagnostic tests (laboratory analyses, abdominal ultrasonography, radiography, endoscopy), removal technique (endoscopic, surgical, manual removal from the rectum, and/or spontaneous expulsion), complications, and follow-up information. Based on the month of presentation, cases were categorized according to the four seasons (autumn, winter, summer, and spring). Remote medical history was reviewed specifically to investigate the presence of chronic gastrointestinal disease suggestive of chronic inflammatory enteropathy (CIE), whereas recent anamnesis was evaluated to identify potential causes of gastric hyperacidity. Ultrasonographic examinations were performed using a Samsung V8 and HM70 system equipped with a micro convex probe (CA4-10M) and ultrasonographic findings were obtained through review of digital medical records and analysis of stored images and video recordings. Additionally, data on the location of GIP were collected through evaluation of surgical and endoscopic reports, as well as review of endoscopic video recordings. The differing compositions of the GIPs analyzed in this study enable their classification into four distinct groups: grass, vegetables, fruit, and other plant material. Based on their location, GIPs were further categorized as gastric, intestinal, or gastrointestinal (for those extending partially from the antrum into the duodenum). When possible, gastric locations were subcategorized as fundus, body, or antrum, while intestinal locations were classified as duodenum, jejunum, ileum, colon, or rectum, based on endoscopic and surgical findings.

Only descriptive statistics are provided. Results are presented as median (range) for continuous variables and as number (percentage) for qualitative and semi-quantitative variables.

## 3. Results

### 3.1. Animals

Eighteen of the 25 animals diagnosed with GIP and considered eligible for the study were included in the analysis. Seven of 25 animals were excluded because the FB consisted of plastic mixed with fibrous material. Seventeen dogs (94.4%) and 1 cat (5.6%) were included in the study. The signalment are stratified in the in Table 1. Most dogs in this study were mixed-breed (5/17, 29.4%), followed by Labrador Retriever (4/17, 23.5%), German Shepherd (2/17, 11.8%), and Pointer (2/17, 11.8%).

The mean age of dogs was 50.4 months, and the mean body weight was 21.7 Kg. When stratified by season, among the 18 cases included in the study, 4 presented in winter (22.2%), 6 in spring (33.3%), 2 in summer (11.1%), and 6 in autumn (33.3%).

### 3.2. Clinical Findings

Remote medical history was available for 10 out of 18 animals (55.6%). Two animals (11.1%) had a previous diagnosis of CIE. Three additional subjects (16.7%) had a history of chronic gastroenteric clinical signs. Three cases (16.7%) had undergone multiple surgical treatments for ingestion of plastic FBs during their lifetime. Among the remaining animals, one had a history of pruritus and excessive limb licking (5.6%) and the last had experienced gastric dilatation and gastric dilatation–volvulus (GDV) (5.6%). In 11.1% of cases (2/18), the recent medical history indicated that the animals were presented to the VTH because the owners had directly witnessed the ingestion of an FB in one case and a disinfectant in the other.

Among the evaluable animals, 5 subjects (27.8%) exhibited a depressed sensorium, and 1 was reported to be in a comatose state (5.6%). Congested mucous membranes were observed in 6/18 subjects (33.3%), elevated body temperature in 4/18 (22.2%), tachypnea in 7/18 (38.9%), tachycardia in just one subject (5.6%) and lymphadenomegaly was detected in two dogs (11.1%). Clinical evidence of dehydration was noted in 3/18 animals (16.7%), based on the presence of dry mucous membranes, delayed skin tent time, and enophthalmos. An altered body condition score (BCS) was recorded in six animals (33.3%): four were underweight (BCS range 2–4), and two were overweight (BCS range 7–8). Abdominal pain was identified in 9/18 cases (50%). In 2/18 animals (11.1%), abdominal palpation revealed a tubular thickening in the pyloric/duodenal region. Lingual erosion was documented in one case (5.6%). Four animals (22.2%) showed cutaneous and subcutaneous alterations, including a dull coat (2/18, 11.1%), cutaneous excoriation (1/18, 5.6%), cutaneous nodules (1/18, 5.6%), and an umbilical hernia (1/18, 5.6%).

Gastrointestinal clinical signs were documented in 88.9% of cases (16/18) and are reported in Table 2. Non-specific clinical signs were observed in 2 of the 18 subjects (11.1%) including pruritus and excessive limb licking (1/18, 5.5%) and depressed sensorium (7/18, 38.8%).

### 3.3. Diagnostic Findings

**Figure 1:**
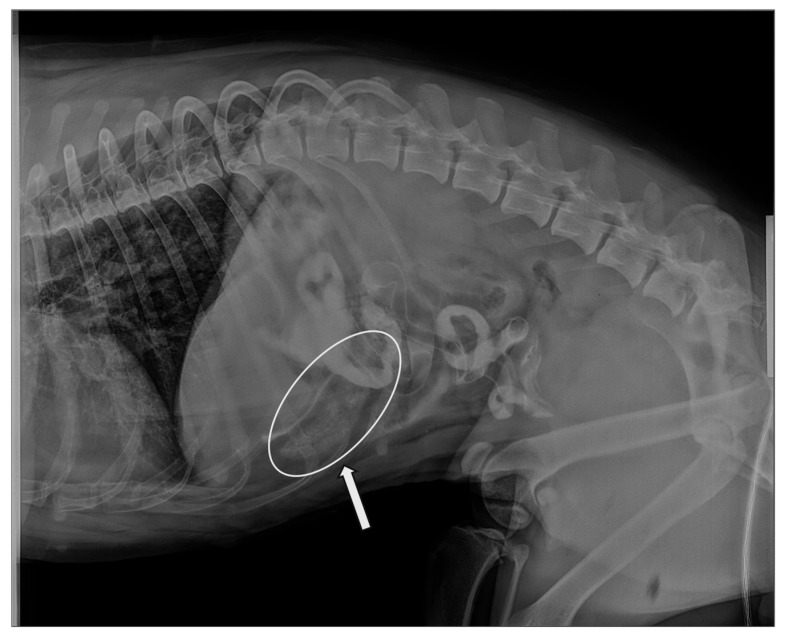
A latero-lateral abdominal radiographic projection with contrast agent showed a dilated segment of small intestine containing radiographically heterogeneous, filamentous material with a grass-like appearance, as indicated by the circle and the arrow.

In 6 of 18 cases (33.3%) included in the present study, complete blood count (CBC) and clinical biochemistry results were unavailable, as these tests had been performed by the referring veterinarians. Diagnostic examinations performed at the VTH included ultrasonography (10/18, 55.6%), radiography (7/18, 38.9%), endoscopy (4/18, 22.2%), gastrointestinal histopathology (2/18, 11.1%), serology (3/18, 16.7%), coprological examination (1/18, 5.6%), and fine-needle aspiration (FNA) from cutaneous nodules (1/18, 5.6%). CBC and biochemistry findings included leukocytosis in 5/18 cases (27.8%), neutrophilia in 4/18 (22.2%), lymphocytopenia in 2/18 (11.1%), increased aspartate aminotransferase and alanine aminotransferase (AST and ALT) in 4/18 (22.2%), increased alkaline phosphatase (ALP) in 1/18 (5.6%), elevated creatinine and urea in 1/18 (5.6%), decreased total protein and albumin in 1/18 (5.6%), elevated creatine phosphokinase (CPK) in 2/18 (11.1%), and hyperglycemia in 1/18 (5.6%). Cytologic evaluation of cutaneous nodules obtained via FNA revealed a neoplastic process consistent with a diagnosis of mast cell tumor. Radiographic reports were available for 6 of 18 cases (33.3%). Radiopaque FBs were identified in 3/6 animals (50%). One subject (1/6, 16.7%) showed a dilated small intestinal segment containing radiographically heterogeneous, filamentous material with a grass-like appearance (Figure 1). Diffuse gaseous distension of the gastrointestinal tract was observed in 3/6 cases (50%), associated with marked overdistension of bowel loops and partial intraluminal fluid and semisolid content. One subject (1/6, 16.7%) showed marked gastric fold thickening and gastric dilation with fluid content.

**Figure 2:**
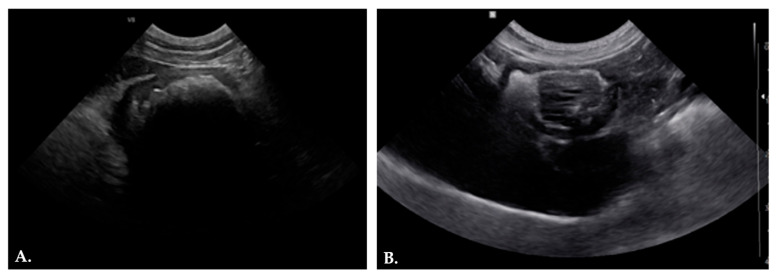
Ultrasonographic features of GIPs. (**A**) Abdominal ultrasonography showed a well-defined intragastric hyperechoic structure associated with a marked posterior AS and signs of peritoneal reactivity, consistent with a gastric FB; (**B**) Abdominal ultrasonography revealed a space-occupying hypoechoic structure within the gastric lumen, characterized by parallel hyperechoic linear striations and surrounded by hyperechoic gastric contents.

Nine out of 18 animals (50%) with suspected gastroenteric obstruction were evaluated by abdominal ultrasonography. Ultrasonographic findings were stratified in Table 1 and varied according to the location and nature of the FB, with evidence of both direct and indirect signs of obstruction. The most frequent ultrasonographic finding was the presence of an intraluminal acoustic shadow (AS), detected in 7/9 cases (77.8%) (Figure 2A). The AS was localized at the gastric level (2/7, 28.6%), within the intestinal tract (3/7, 42.9%), or extended along multiple segments of the gastrointestinal tract (2/7, 28.6%). In one dog (14.3%), the AS extended from the stomach through the small intestine up to the ileo-cieco-colic valve. In one case (14.3%) the gastric FB presented a distinctive ultrasonographic appearance: a space-occupying, irregular, hypoechoic structure with parallel hyperechoic striations, surrounded by hyperechoic ingesta (Figure 2B).

Gastric abnormalities were observed in 4/9 cases (44.4%) and included gastric hypomotility, fluid accumulation, and gastric wall thickening, consistent with nonspecific gastric distress. In 1/9 subject (11.1%), ultrasonographic findings were compatible with gastritis, characterized by redundancy of gastric folds and increased mucosal echogenicity. Intestinal loop dilation was observed in 3/9 cases (33.3%), mainly involving the jejunum and ileum, and in 1/9 animal (11.1%) severe and diffuse dilation of the intestinal loops was detected. Hypomotility of the gastrointestinal tract was observed in 5/9 cases (55.6%). Signs of peritoneal reactivity, characterized by increased echogenicity of the mesenteric fat, were identified in 3/9 cases (33.3%), particularly in dogs with severe gastrointestinal hypomotility. An intestinal intussusception was diagnosed in 1/9 animal (11.1%), characterized by the typical concentric multilayered appearance of the intestinal walls, associated with an intraluminal AS and marked dilation of the proximal intestinal loops.

### 3.4. GIP Type and Therapeutic Management

The GIPs consisted of grass aggregates (11/18, 61.1%), vegetables (1/18, 5.6%), fruit (1/18, 5.6%), and non-specific plant material (5/18, 27.8%) including hemp fibers in one case. Of the 18 animals, 3 underwent endoscopic removal (16.7%), 7 required surgical treatment (38.9%), in 2 cases (11.1%) the GIPs were manual retrieved from rectal ampulla, and 6 expelled the GIPs spontaneously through defecation (33.3%). The GIP composition was confirmed by direct visualization of grass or other plant material during endoscopic inspection in 8 of 18 cases (44.4%), whereas in the 7 animals (38.9%) that underwent surgical intervention, the diagnosis was confirmed intraoperatively by the surgeon following GIP removal. In 2 dogs (11.1%), the GIPs were retrieved from the rectal ampulla, and our vegetal nature were confirmed by the attending veterinarian. The only feline subject (5.6%) expelled a grass mass via defecation, which was subsequently submitted by the owners to the veterinarian at the VTH for evaluation and was confirmed as a GIP.

Localization was gastric in 7/18 cases (38.9%), involving the antrum in 6 animals and the fundus of the stomach in one. Intestinal localization was observed in 7/18 animals (38.9%): four in the jejunum (22.2%), one at the ileocecal valve (5.6%), and two in the rectal ampulla (11.1%). In 2/18 cases (11.1%), the GIP extended from the pylorus into the duodenum and was therefore classified as gastrointestinal. In the remaining 2/18 subjects (11.1%), the GIPs were expelled in the feces, transiting through the entire gastrointestinal tract without inducing any apparent clinical signs.

Endoscopic examination (gastroscopy) was performed in 8 out of 18 cases (44.4%). Among these, mechanical fragmentation and/or removal of the GIP were successfully achieved in 3 subjects (16.7%) using grasping and roth-tooth forceps. In one case (5.6%), attempts at endoscopic removal of the GIP were unsuccessful. In 4 out of 18 animals (22.2%), the GIPs was identified incidentally. Specifically, one GIP was detected during diagnostic endoscopy performed for suspected CIE; another was identified during endoscopic evaluation of mucosal lesions secondary to disinfectant ingestion; and 2 additional cases underwent endoscopy due to suspected plastic FBs based on the history provided by the owners and referring veterinarians. Because the GIPs were discovered incidentally, they were left in the stomach, and the clinical condition of the animals was subsequently monitored. Histopathological analyses were performed on gastric and intestinal biopsies from 2 dogs (11.1%), including one with a polypoid lesion in the pyloric antrum. In the other case, endoscopic biopsies were obtained as part of the standardized diagnostic workup for dogs presenting with CIE.

Surgical intervention was undertaken in 7 of 18 subjects (38.9%). In 5 dogs (27.8%), the GIP was confined to the small intestine, warranting removal via enterotomy. In the remaining 2 cases (11.1%), the GIP extended from the stomach into the small intestine, and a combined gastrotomy–enterotomy approach was required. In 2 subjects (11.1%), removal of the GIP required digital rectal extraction, as the mass had become impacted within the rectal ampulla. The remaining 6 animals (33.3%) spontaneously expelled the GIP in their feces.

Follow-up data were available for 16 of 18 cases (88.9%). All monitored animals showed progressive clinical improvement after GIP removal. No complications were recorded in 17 of 18 subjects (94.4%). One animal (5.6%) developed postoperative enteritis, which resolved within one week following supportive symptomatic medical therapy.

## 4. Discussion

This retrospective study analyzed the clinical presentation, diagnostic findings, and treatment of GIP in small animals. To the best of our knowledge, no similar studies have been previously reported in veterinary literature. Most animals with GIP presented with acute gastrointestinal signs and had a history of, or were suspected to have, CIE. Treatment approaches included endoscopic removal, surgical intervention, or manual retrieval; however, in some cases, spontaneous resolution occurred without medical intervention.

A detailed analysis of recent and past medical histories was performed for all GIP cases to identify potential predisposing factors, both on an individual and population basis. In humans, predisposing factors include gastric emptying disorders, impaired pyloric motility, previous partial gastrectomy, high-fiber diets, and antisecretory therapies, which induce hypochlorhydria and reduce digestive enzyme activity (e.g., pepsin and cellulase) [13]. Review of remote medical history revealed that two animals had a prior diagnosis of CIE, while three others, though undiagnosed, had experienced gastrointestinal signs during their lifetime. In individuals affected by CIE, the inflammatory process involving gastric and intestinal mucosa is often characterized by recurrent exacerbations [14]. These inflammatory flare-ups are commonly associated with the development of acute gastroenteric clinical signs, such as nausea, pica, and gastroesophageal reflux, which may contribute to an increased tendency to ingest plant material, including grass [15]. At the same time, the persistent underlying inflammation may impair normal digestive function and gastrointestinal motility, leading to delayed transit and abnormal peristalsis. This altered gastrointestinal environment may promote the accumulation and retention of ingesta, thereby predisposing affected animals to the formation of GIPs, as demonstrated in human medicine [14,15,16]. Evidence supporting this pathogenetic mechanism in veterinary medicine comes from the predisposition of cats with CIE to develop trichobezoars and, in some cases, gastrointestinal obstruction caused by their presence [17,18]. Another element suggesting that CIE may be a risk factor for the development of GIP is the predominance of Labrador Retrievers and German Shepherds in this study, representing 22.2% and 11.1% of cases, respectively. Notably, Kathrani et al. (2011) demonstrated that certain canine breeds, including Labrador Retrievers and German Shepherds, show a higher susceptibility to the development of CIE [19]. However, the limited sample size and the absence of comparative statistical analysis prevent any definitive conclusions, and these findings may simply reflect the demographics of the local population.

One dog experienced both gastric dilation and GDV. Episodes of gastric dilation, especially when complicated by GDV, can result in persistent functional and structural changes in the stomach, including impaired gastric motility, delayed gastric emptying, alterations in gastric innervation (particularly of the vagus nerve) and anatomical modifications following gastropexy [20]. These changes may predispose affected individuals to chronic or recurrent gastrointestinal signs. Additionally, as previously described in human medicine, impaired gastrointestinal motility can lead to retention of gastric contents, dysbiosis, and, in some cases, the formation of bezoars [21,22].

Based on recent medical history, one case had been diagnosed with cutaneous mastocytoma. There is evidence suggesting a connection between mast cell tumors and gastrointestinal inflammation in dogs, mediated by the biological activity of mast cells [23]. Mast cells contain and release a variety of bioactive mediators, including histamine, that can influence gastrointestinal physiology, potentially leading to gastric hyperacidity and mucosal irritation (e.g., through H2-mediated stimulation of gastric acid secretion) and contributing to gastrointestinal signs such as vomiting, diarrhea, anorexia, and even ulceration [23]. The recent medical history of another dog indicated that the animal was presented to the VTH after the owners directly witnessed the ingestion of a disinfectant. The ingestion or exposure to household cleaning products and disinfectants can induce gastrointestinal irritation, vomiting, abdominal pain, diarrhea, and, in some cases, deeper mucosal lesions [24]. These symptoms may prompt the animal to ingest large amounts of indigestible material, such as grass, as an attempt to relieve the discomfort caused by gastric irritation. This behavior, combined with dysbiosis, impaired gastrointestinal motility, and altered digestion, can contribute to the development of a GIP.

In humans, the prevalence of bezoar detection during upper gastrointestinal endoscopy is approximately 0.5%, with GIP representing the most common type of bezoar [4]. Few data are available in veterinary medicine regarding the prevalence of GIP. This condition has previously been described as uncommon in horses and small ruminants, while, to the best of the authors’ knowledge, information on its frequency in dogs and cats is not available [6,7,25]. The proportion of GIP cases managed by endoscopic removal relative to the total number of FBs treated endoscopically was, on average, 3.15%. This estimate was derived from a comparison with a previously published study by Maggi et al. (2023) conducted at the same institution, which evaluated endoscopic FB removal over a comparable time frame [26]. These findings suggest that the prevalence of this condition is exceedingly low. Nevertheless, comparable data regarding GIP cases managed surgically or those resolving through spontaneous passage are currently unavailable, thereby limiting broader epidemiological comparisons. Another notable finding of our study is that GIP appears to be more frequent in dogs than in cats, with only one feline case observed in this series. This difference may be related to environmental factors, such as lower exposure to indigestible fibers in domestic settings, and/or species-specific behaviors, with grass ingestion as a behavioral or pica-related habit being more pronounced in dogs than in cats.

Although bezoars are relatively rare and often incidental findings, they can cause non-specific clinical signs such as nausea and a feeling of fullness. When complicated, they may lead to gastritis, gastric ulceration or perforation, gastric obstruction, or small intestinal obstruction [27,28]. In the present study, the most common gastrointestinal clinical signs were vomiting (44.4%), abdominal pain (44.4%), and decreased appetite (38.9%). These clinical signs may be associated with the presence of GIP and the resulting secondary gastrointestinal obstruction, as suggested by the high number of animals requiring endoscopic or surgical management. Alternatively, they may be secondary to gastrointestinal inflammation or represent a manifestation of an underlying gastrointestinal disease. Non-specific clinical signs observed in some cases, such as depression, ataxia, tachycardia, and tachypnea, may be associated with dehydration from gastrointestinal losses [29]. In dogs and cats, pica is defined as the recurrent ingestion of non-food materials and may be associated both with behavioral disorders and with underlying medical conditions, such as CIE [30]. The behavioral basis of pica remains incompletely understood, stress, environmental factors, and compulsive tendencies (particularly in younger animals) have been implicated [31]. In this study three of the 18 animals (16.7%) exhibited pica, and 27.8% (5/18) had a history of FBs ingestion. These findings suggest that pica may represent both a behavioral manifestation and a clinical indicator in animals predisposed to phytobezoar formation, linking abnormal ingestive behavior with underlying gastrointestinal pathology and underscoring the need for thorough clinical assessment of affected individuals. Regarding seasonality, the highest frequency of presentations was observed in spring and autumn, each accounting for 33.3% of cases. This contrasts with our hypothesis, which relates the higher occurrence to the increased lignin content in vegetable fibers during the drier months. Considering the association between gastrointestinal signs and gastrointestinal impaction, whether primary or secondary to phytobezoar ingestion, a potential relationship between GIP occurrence and the seasonal distribution of other gastrointestinal disorders was hypothesized. However, current veterinary literature provides limited data on the prevalence and seasonal distribution of gastrointestinal clinical signs in small animals, with some studies identifying winter as the season of highest incidence, which contrasts with the distribution observed in our results [32].

Laboratory findings in cases of GIP often reflect secondary inflammatory processes or dehydration resulting from gastrointestinal losses. Most animals exhibited leukocytosis, neutrophilia, and lymphocytopenia, consistent with a stress leukogram [33]. In some subjects, elevated transaminase levels (AST, ALT) may indicate reactive hepatitis secondary to acute gastroenteropathy [34]. Conversely, increased ALP activity could be related to reduced intestinal motility and subsequent biliary stasis, likely associated with the dysorexia or anorexia observed in two dogs in our study [35]. Elevated creatinine and urea levels are likely attributable to severe dehydration [36]. Hypoproteinemia and hypoalbuminemia in one animal may reflect worsening gastroenteritis secondary to the GIP. While hypoalbuminemia alone generally indicates an acute-phase inflammatory response (as albumin is a negative acute-phase protein), concurrent loss of total protein fractions is typically associated with protein-losing enteropathy (PLE), a specific form of CIE [37,38].

In accordance with other previous studies, abdominal radiography and ultrasonography are commonly used as first-line diagnostic tools, but both modalities have notable limitations [39,40]. Radiographic findings could not be specifically attributed to the presence of a GIP and were instead consistent with nonspecific features commonly associated with FBs. In only one case (5.6%), radiographic examination of a small intestinal segment revealed heterogeneous, filamentous material with a grass-like appearance (Figure 1). However, the absence of characteristic radiographic patterns of GIP in the remaining animals, precluded the identification of a consistent model capable of distinguishing these lesions from other types of gastrointestinal FBs. Ultrasonography remains a valuable first-line tool for the evaluation of gastrointestinal obstruction in small animals, allowing detection of both direct signs, such as intraluminal AS, and indirect indicators, including bowel dilation, hypomotility, and peritoneal reactivity. In our series, AS were observed in the majority of cases, particularly in the stomach and proximal intestine, highlighting the modality’s utility in identifying FB. Distinguishing bezoars from other types of FBs, however, can be challenging [11]. For instance, a gastric bezoar may appear with irregular contours and internal shadows that mimic foreign material but are often less distinct than harder objects, such as stones or bones. Similar diagnostic difficulties occur with soft FBs, including fabric or socks, which may produce subtle or variable ultrasonographic patterns [12]. Notably, distinctive features, such as the hypoechoic structure with parallel hyperechoic striations observed in a fibrous vegetable FB in our series (Figure 2B), can aid in characterizing the nature of ingested material and guide clinical management.

Therapeutic management of GIP often requires medical intervention. In fact, among the 18 animals included in the study, 38.9% required surgical treatment, 16.7% underwent endoscopic removal, and 11.1% had the GIP manually retrieved from the rectal ampulla. Only 33.3% of the animals expelled the GIP spontaneously through defecation. Gastrointestinal FBs represent common surgical emergencies in small animal medicine and may present with a wide range of clinical signs depending on the location, severity, and duration of the obstruction [26,28]. All types of bezoars have the potential to cause gastric obstruction or, when located more distally, intestinal obstruction, including cases of GIPs [28]. In contrast to human medicine, where only 0.4–4.8% of GIPs cause intestinal obstruction, most animals in our study required surgical intervention for this reason [4]. In the present study, 6 out of 18 animals underwent primary surgical management, consisting of gastrotomy and/or enterotomy. In each of these animals, surgical therapy was the treatment of first choice, as the GIP had already caused a jejunal intestinal obstruction, rendering an endoscopic approach no longer applicable. One dog initially underwent endoscopic treatment because the GIP extended from the pyloric antrum to the distal portion of the duodenum; partial endoscopic fragmentation of the vegetal mass was achieved. However, the endoscopic approach proved unsuccessful, and surgical intervention was subsequently required. Moreover, two animals required surgical intervention consisting of gastrotomy and enterotomy, as the bezoar was firmly impacted within the pyloric antrum and extended distally into the duodenum and the proximal portion of the jejunum. In veterinary medicine, a standardized scientific definition for this specific type of gastrointestinal obstruction has not yet been established. In contrast, in human medicine, gastric obstruction caused by a trichobezoar with a tail extending into the duodenum, jejunum, or beyond the ileocecal valve is defined as “rapunzel syndrome” [1,10]. Authors suggest considering surgery as the first-line therapeutic approach in cases of intestinal obstruction caused by GIP. In our study, the diagnosis was established through imaging, which revealed dilated intestinal loops associated with intraluminal AS, consistent with an FB, in the clinical context of acute abdomen. When the obstruction also involves the gastric component (such as in cases of linear FBs, or, as in our case, a FB extending from the pylorus to the duodenum) an endoscopic approach may be attempted. However, in cases of failure, surgical management remains essential.

**Figure 3:**
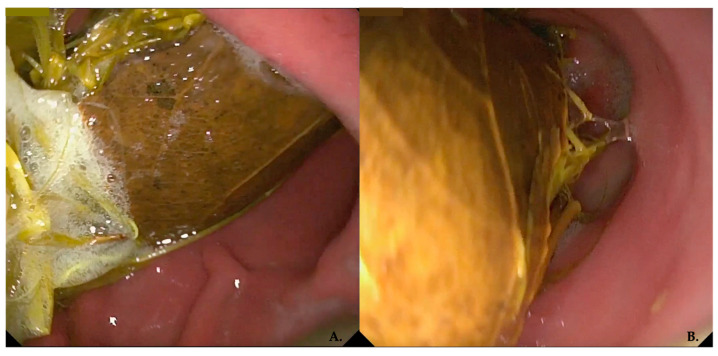
Endoscopic findings of antral obstruction by GIP. (**A**,**B**) refer to the same animal and illustrate the presence of a GIP lodged in the pyloric antrum, resulting in a obstruction of the gastric outlet.

Of the 8 cases that underwent endoscopic examination of the stomach, ultrasonography had identified only two GIP cases prior to endoscopy. This indicates that, in the majority of animals, the detection of the GIP was entirely incidental, as the animals presented with nonspecific gastrointestinal signs or had been referred to our department for unrelated reasons, such as suspected FB ingestion and/or disinfectant ingestion. Mechanical fragmentation and/or endoscopic removal of the GIPs were successfully achieved in 3/18 subjects using grasping forceps and roth-tooth forceps. This technique facilitates the fragmentation of the GIP, thereby reducing its size and enhancing its exposure to digestive enzymes, ultimately promoting its dissolution [39]. In human medicine, medical management through enzymatic disintegration is most effective on GIPs, in which plant fibers are susceptible to dissolution [40]. The most commonly reported substance used is dark soda (e.g. Coca-Cola, RC Cola, Pepsi), where the acidity from carbonic and phosphoric acid allows for fiber digestion, the sodium bicarbonate acts as a mucolytic, and carbon dioxide bubbles penetrate between fibers to increase the surface area for interaction with acid. In some cases, medical therapy can soften the consistency of the bezoar, making it more manageable during endoscopic or surgical procedures [2,40,41]. In our study, endoscopically diagnosed GIPs were located in the pyloric antrum and resulted in obstruction in two cases (Figure 3); in only one dog the GIP was located in the gastric fundus.

Enzymatic disintegration was not adopted as a management strategy because prompt removal of the GIP was required due to the presence of antral obstruction, which could lead to metabolic alkalosis [42,43]. Successful treatment was achieved through endoscopic fragmentation and removal of the fibrous material, rendering the administration of enzymatic agents unnecessary. In a case of GIP involving the gastric fundus, endoscopic removal was performed despite the lack of clinical signs, as ultrasonographic findings suggested persistence of the FB for approximately two weeks. In this dog, a concomitant polypoid lesion in the pyloric antrum (later diagnosed as an adenomatous polyp) was identified. This finding may further support the hypothesis that a chronic inflammatory state can impair gastric digestion and represent a risk factor for GIP formation. Endoscopic examination proved to be a key tool in the diagnosis of GIPs and, in some cases, in their therapeutic management, allowing direct fragmentation and removal of the GIP. In two animals, the GIP passed beyond the ileocecal valve (considered a critical narrowing for intestinal FBs and a common site of obstruction) and subsequently became impacted in the rectal ampulla, resulting in constipation [44]. Constipation in these cases may have been exacerbated by factors such as excessive dietary fiber intake, which can increase fecal bulk and promote impaction, particularly in the presence of poorly digestible material [45]. In both cases, the GIP was manually removed, leading to resolution of the pathological condition. In 6 out of 18 animals, the detection of GIP was entirely incidental. In four of these cases, the GIP was located in the stomach (specifically in the pyloric antrum) and was identified during gastroscopy performed for unrelated reasons. However, neither endoscopic nor surgical intervention was deemed necessary, as these animals did not exhibit acute clinical signs of gastrointestinal obstruction. When GIPs cause gastrointestinal obstruction, they may disrupt fluid balance, acid–base homeostasis, and electrolyte levels as a result of fluid sequestration, vomiting, and reduced oral intake. Complete obstruction is typically associated with acute clinical signs and rapid clinical deterioration, whereas partial obstruction may lead to chronic manifestations such as maldigestion and malabsorption [11,46]. For this reason, careful clinical monitoring of affected animals is essential. Nevertheless, in the present cases, the animals were closely monitored, and the GIP passed spontaneously through the gastrointestinal tract and was ultimately expelled in the feces. Follow-up dates demonstrated progressive clinical improvement after removal or natural transit of the GIP. No complications were observed in 17 of 18 animals (94.4%). One subject developed postoperative enteritis, which resolved within one week with supportive symptomatic medical therapy. Based on the case series presented in this study, endoscopic and/or surgical removal is indicated in GIP cases presenting with acute gastrointestinal symptoms and obstruction. Accordingly, an endoscopic approach should be considered in cases of refractory vomiting, clinical signs suggestive of pyloric obstruction confirmed by endoscopic evaluation, and/or persistent sonographic findings consistent with a gastric FB. Surgical removal, conversely, is indicated in all cases of intestinal obstruction when endoscopic management is no longer feasible.

The limitations of this study are related to its retrospective design, small sample size, and lack of a control group. The limited number of cases reduces statistical power and precludes any conclusions regarding correlations between CIE and GIP. Moreover, the retrospective nature of the study results in reliance on pre-existing clinical data, which are often heterogeneous or incomplete, with a possible lack of standardization in diagnostic, therapeutic, and follow-up protocols. A further limitation of this study is the inclusion of only a single feline case. This observation may reflect the lower prevalence of GIPs in cats; however, the small sample size precludes any statistical analysis to substantiate this hypothesis. In humans, most GIPs are asymptomatic, and in several cases in our series, the diagnosis was incidental. It should be noted that animals affected by GIPs that do not exhibit clinical signs or complications are unlikely to present to veterinary centers and are therefore underrepresented in the available data. This inherent limitation may have resulted in an overestimation of the prevalence of GIPs requiring endoscopic or surgical intervention and should be carefully considered when interpreting the findings of this study.

## 5. Conclusions

GIPs in dogs and cats are currently considered an uncommon condition, and the existing veterinary literature provides limited information on this topic. This article emphasizes the clinical relevance of GIP in small animals, providing an overview of their diagnostic approaches and therapeutic management. In suspected GIP cases, careful ultrasonographic evaluation for acoustic shadowing, combined with a thorough medical history, can enhance diagnostic accuracy. Endoscopy may confirm the diagnosis and, if necessary, permit treatment via mechanical fragmentation and/or removal. For GIPs located in the intestine, or involving both the stomach and intestine with signs of gastrointestinal obstruction, surgical intervention should be regarded as the first-line treatment. Further prospective, comparative, large-scale, and multicenter studies involving subjects diagnosed with GIP are warranted to better assess the factors influencing their development, particularly the correlation between CIE and GIP. Such studies are also necessary to establish evidence-based therapeutic guidelines, similar to those available in human medicine [3].

## Figures and Tables

**Table 1:** This Table summarizes the signalment, diagnostic findings, and treatment of the cases included in this study. Signalment data are stratified by species, age, sex, and breed.

Animals	Signalment	Ultrasonography Findings	Radiological Findings	Treatment or Resolution
Case 1	Dog, Mixed-breed, NF, 6 y, 15.45 Kg	AS extending from the stomach through the proximal duodenum GI hypomotility	-	Endoscopic fragmentation
Case 2	Dog, Mixed-breed, NM, 8 y, 33 Kg	-	Diffuse and marked distension of the intestinal loop	Defecation
Case 3	Dog, Mixed-breed, F, 5 y, 6 Kg	Gastric AS	-	Endoscopic fragmentation
Case 4	Dog, Belgian Sheperd, NF, 2 y, 23 Kg	-	-	Defecation
Case 5	Dog, German Sheperd, M, 4 y, 28 Kg	-	-	Defecation
Case 6	Dog, Labrador Retriever, F, 5 y, 27 Kg	-	-	Defecation
Case 7	Dog, Pinscher, F, 8 y, 4 Kg	Gastric FB characterized by irregular hypoechoic structure with parallel hyperechoic striations	-	Endoscopic removal
Case 8	Dog, American Staffordshire Terrier, NF, 4 y, 22 Kg	Intestinal AS Diffuse dilation of the intestinal loops PR	Dilated small intestinal segment containing a grass-like appearance	Enterotomy
Case 9	Dog, Pointer, F, 2 y, 19 Kg	-	Diffuse and marked distension of the intestinal loop	Enterotomy
Case 10	Dog, American Pit bull, M, 5 y, 29.7 Kg	Gastric hypomotility AS localized through the ileocecocolic valve Abdominal free fluid PR	-	Enterotomy
Case 11	Dog, Labrador Retriever, M, 4 y, 29.3 Kg	-	-	Enterotomy
Case 12	Dog, Labrador Retriever, F, 6 y, 24.5 Kg	AS in the jejunum Intestinal hypomotility	Diffuse and marked distension of the intestinal loops	Enterotomy
Case 13	Dog, Mixed-breed, M, 5 y, 20 Kg	-	Diffuse and marked distension of the intestinal loops Radiopaque intestinal FB	Manual retrieval from the rectal ampulla
Case 14	Dog, Mixed-breed, F, 9 y, 35 Kg	No pathological findings	-	Manual retrieval from the rectal ampulla
Case 15	Dog, German Sheperd, F, 5 y, 27 Kg	Gastric wall thickening with increased mucosal echogenicity Redundancy of gastric folds	-	Defecation
Case 16	Dog, Labrador Retriever, M, 9 y, 24.8 Kg	-	Radiopaque gastrointestinal FB Marked thickening of the gastric folds Gastric dilation	Gastrotomy and Enterotomy
Case 17	Dog, Pointer, M, 6 y, 29.5 Kg	AS within the stomach extending through the entire small intestine to the ileocecocolic valve Gastric wall thickening Severe intestinal loop dilation Intussusception	-	Gastrotomy and Enterotomy
Case 18	Cat, Mixed-breed, M, 1 y, 4.1 Kg	-	-	Defecation

AS: Acoustic Shadow; FB: Foreign Body; GI: Gastrointestinal; NF: Neutered Female; NM: Neutered Male; PR: Peritoneal Reactivity.

**Table 2:** The table shows the frequency and percentage of gastrointestinal clinical signs observed in the cases included in this study.

Gastrointestinal Clinical Signs	Frequency	Percentage (%)
Vomiting	8/18	44.4%
Loss of appetite	7/18	38.9%
Diarrhea	4/18	22.2%
Grass-seeking behavior Pica	3/18	16.7%
Weight loss Repeated licking Empty swallowing movements	1/18	5.6%

## Data Availability

The data present in this study is available on request from the corresponding author due to privacy and legal reasons.

## Abbreviations

The following abbreviations are used in this manuscript:

ALP	Alkaline phosphatase
ALT	Alanine aminotransferase
AS	Acoustic Shadow
AST	Aspartate aminotransferase
BCS	Body condition score
CBC	Complete blood count
CIE	Chronic inflammatory enteropathy
CPK	Creatine phosphokinase
FB	Foreign body
GDV	Gastric dilatation-volvulus
GI	Gastrointestinal
GIP	Gastrointestinal phytobezoar
NF	Neutered Female
NM	Neutered Male
PLE	Protein-losing enteropathy
PR	Peritoneal Reactivity
VTH	Veterinary teaching hospital
